# Biotransformation of ferulic acid to protocatechuic acid by *Corynebacterium glutamicum* ATCC 21420 engineered to express vanillate *O*-demethylase

**DOI:** 10.1186/s13568-017-0427-9

**Published:** 2017-06-21

**Authors:** Naoko Okai, Takaya Masuda, Yasunobu Takeshima, Kosei Tanaka, Ken-ichi Yoshida, Masanori Miyamoto, Chiaki Ogino, Akihiko Kondo

**Affiliations:** 1 Graduate School of Science, Technology and Innovation, Kobe University, 1-1 Rokkodaicho, Kobe, 657-8501 Japan; 2 Raw Materials and Polymers Division, Raw Materials and Polymers Technology Department, Teijin Limited, 2345 Nishihabu-cho, Matsuyama, Ehime 791-8536 Japan; 3 Organization of Advanced Science and Technology, Kobe University, 1-1 Rokkodaicho, Kobe, 657-8501 Japan; 4 Department of Chemical Science and Engineering, Graduate School of Engineering, Kobe University, 1-1 Rokkodaicho, Kobe, 657-8501 Japan; 5 Biomass Engineering Program, RIKEN, 1-7-22 Suehiro-cho, Tsurumi-ku, Yokohama, 230-0045 Japan

**Keywords:** *Corynebacterium glutamicum*, Protocatechuic acid, Ferulic acid, Biotransformation

## Abstract

Ferulic acid (4-hydroxy-3-methoxycinnamic acid, FA) is a lignin-derived phenolic compound abundant in plant biomass. The utilization of FA and its conversion to valuable compounds is desired. Protocatechuic acid (3,4-dihydroxybenzoic acid, PCA) is a precursor of polymers and plastics and a constituent of food. A microbial conversion system to produce PCA from FA was developed in this study using a PCA-producing strain of *Corynebacterium glutamicum* F (ATCC 21420). *C. glutamicum* strain F grown at 30 °C for 48 h utilized 2 mM each of FA and vanillic acid (4-hydroxy-3-methoxybenzoic acid, VA) to produce PCA, which was secreted into the medium. FA may be catabolized by *C. glutamicum* through proposed (I) non-β-oxidative, CoA-dependent or (II) β-oxidative, CoA-dependent phenylpropanoid pathways. The conversion of VA to PCA is the last step in each pathway. Therefore, the vanillate *O*-demethylase gene (*van*AB) from *Corynebacterium efficiens* NBRC 100395 was expressed in *C. glutamicum* F (designated strain FVan) cultured at 30 °C in AF medium containing FA. Strain *C. glutamicum* FVan converted 4.57 ± 0.07 mM of FA into 2.87 ± 0.01 mM PCA after 48 h with yields of 62.8% (mol/mol), and 6.91 mM (1064 mg/L) of PCA was produced from 16.0 mM of FA after 12 h of fed-batch biotransformation. Genomic analysis of *C. glutamicum* ATCC 21420 revealed that the PCA-utilization genes (*pca* cluster) were conserved in strain ATCC 21420 and that mutations were present in the PCA importer gene *pca*K.

**Electronic supplementary material:**

The online version of this article (doi:10.1186/s13568-017-0427-9) contains supplementary material, which is available to authorized users.

## Introduction

Ferulic acid (4-hydroxy-3-methoxycinnamic acid, FA) is abundant in nature and is derived from lignocellulosic biomass. FA is esterified to an arabinosyl residue of arabinoxylan in the plant cell wall and is linked to lignin (Walton et al. 2000). FA is a 3-*o*-methyl phenolic moiety of lignin that crosslinks hemicellulose and lignin in the plant cell wall (Gopalan et al. 2015). FA is present in abundance in crops and in agricultural wastes such as rice bran, maize bran, corn and wheat brans, or straw (Gopalan et al. 2015). FA can be released from arabinosyl residues of arabinoxylan in the plant cell wall using alkaline solvents or enzymes (Gopalan et al. 2015). Maize bran contains approximately 30 g FA/kg dry weight of cell wall materials (Benoit et al. 2006). Corn and rice bran each contain approximately 32 and 33 g FA/kg dry weight of cell wall material, respectively (Mathew and Abraham 2004; Schmidt et al. 2013). FA is an inexpensive aromatic derived from plant biomass, which serves as a substrate for bioconversion to high-value aromatics.

Protocatechuic acid (PCA) is a precursor of polymers and plastics. For example, the copolymer of PCA and aniline serves as an electrode with high electrochemical potential (Sun et al. 1998). The PCA isomer 2, 4-dihydroxybenzoic acid consists of core–shell polymer aerogels (Carrott et al. 2012) and carbon aerogel-containing metals (Carrott et al. 2010). PCA is a constituent of fruits and vegetables and has potential as an ingredient of food. PCA is an antioxidant that may prevent human diseases (Masella et al. 2012).

PCA produced from biomass-FA by engineered microorganisms has potential use for the production of biomass-derived plastics and biopharmaceuticals as well as cosmetics and food ingredients. For example, PCA can be synthesized from glucose by *Corynebacterium glutamicum* F (ATCC 21420) that expresses *Escherichia coli* chorismate-pyruvate lyase (CPL) (Okai et al. 2016). The recombinant* C.* *glutamicum* strain FUbiC, which synthesizes PCA from glucose via the chorismate pathway, secretes PCA into the medium (Okai et al. 2016). Further, wild-type *C. glutamicum* (ATCC 21420) grown on glucose produces extracellular PCA (Okai et al. 2016).

*Corynebacterium glutamicum* is a nonpathogenic, nonmotile gram-positive *Actinomycetales* that includes *Rhodococci, Nocardia*, and other related microorganisms (Whitman et al. 2012). *C. glutamicum* is used in industry to produce amino acids such as glutamate and lysine (Hermann et al. 2003). Engineered strains of *C. glutamicum* produce high levels of succinic acid (Okino et al. 2008a) used to synthesize polymers as well as l-lactic acid (Okino et al. 2005) and d-lactic acid (Okino et al. 2008b), which serve as precursors of polylactic acids. Engineered strains of *C. glutamicum* also produce the polymer precursor C5-diamine cadaverine (Tateno et al. 2009) and GABA, which is a precursor of synthetic C4-biodegradable polyamides (Takahashi et al. 2012; Okai et al. 2014). Various “biomass-aromatic plastics” will be synthesized using PCA synthesized from biomass FA by engineered *C. glutamicum*. Therefore, in the present study, we generated an engineered strain of *C. glutamicum* F (ATCC 21420) to produce PCA from FA.

**Fig. 1:**
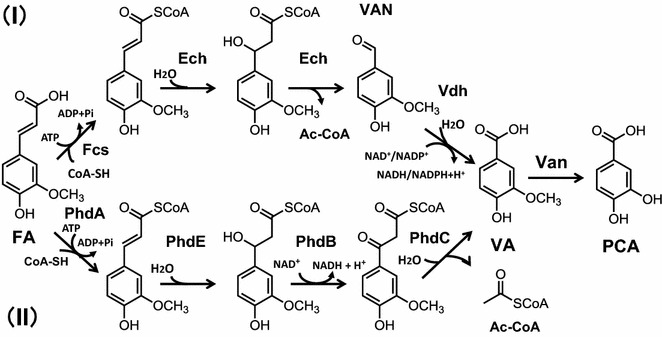
Schematic of two proposed pathways for the biofermentation of FA by *C. glutamicum*. *I* Non-β-oxidative, CoA-dependent catabolic pathway (Merkens et al. 2005; Brinkrolf et al. 2006). *Fcs* feruloyl-CoA synthase, *Ech* enoyl-CoA hydratase/aldolase, *Vdh* vanillin dehydrogenase, and *Van* vanillate 3-*O*-demethylase. *II* β-oxidative, CoA-dependent phenylpropanoid degradation pathway (Kallscheuer et al. 2016). *PhdA* acyl-CoA ligase, *PhdE* enoyl-CoA hydratase, *PhdB* 3-hydroxyacyl-CoA dehydrogenase, *PhdC* 3-oxoacyl-CoA ketohydrolase, and Van

Wild-type *C. glutamicum* (ATCC 13032) grows on FA, vanillin (4-hydroxy-3-methoxybenzaldehyde, VAN), VA, and PCA as carbon sources (Merkens et al. 2005; Shen and Liu 2005). *C. glutamicum* converts FA to PCA via the proposed pathways as follows: (I) non-β-oxidative, CoA-dependent (Merkens et al. 2005; Brinkrolf et al. 2006) and (II) β-oxidative, CoA-dependent degradation of phenylpropanoid (*phd*) (Kallscheuer et al. 2016) (Fig. 1).

(I) In the non-β-oxidative catabolic pathway, FA is transformed to PCA by successive reactions with Fcs, Ech, Vdh, and Van (Merkens et al. 2005; Shen and Liu 2005). In this pathway, FA is activated to feruloyl-CoA by feruloyl-CoA synthase (Fcs, EC 6.2.1.34, encoded by *fcs*). The CoA-thioester of feruloyl-CoA is hydrated and cleaved to VAN and acetyl-CoA by enoyl-CoA hydratase/aldolase (Ech, EC: 4.2.1.17, encoded by *ech*). Vanillin dehydrogenase (Vdh, EC 1.2.1.67, encoded by *vdh*) oxidizes VAN to VA (Ding et al. 2015; Merkens et al. 2005). VA is then catabolized to PCA by vanillate-*O*-demethylase (Van, EC1.14.13.82, encoded by *vanAB*) (Merkens et al. 2005).

(II) FA is metabolized via the phenylpropanoid degradation (Phd) pathway, which yields 3,4-disubstituted benzoic acid and acetyl-CoA from phenylpropanoids such as FA and *p*-coumaric acid, and FA is converted into VA and acetyl-CoA (Kallscheuer et al. 2016). The conversion of VA to PCA is likely the last step of each pathway.

In the present study, production of PCA from FA was achieved using a PCA-producing strain of *C. glutamicum* ATCC 21420 (F). The *C. glutamicum* type strain ATCC 13032 degrades PCA into the TCA cycle intermediates acetyl-CoA and succinyl-CoA via the β-ketoadipate pathway (Shen and Liu 2005; Brinkrolf et al. 2006). In contrast, PCA accumulates in the medium of cultures of strain F (Okai et al. 2016) that therefore shows promise as a robust producer of PCA from FA. Vanillate demethylase (Van, EC 1.14.13.82) catalyzes the 3-*O*-demethylation of VA to produce PCA, which is the last step in the conversion of FA to PCA. The cDNAs encoding vanillate demethylases of *Streptomyces* species (Nishimura et al. 2006) and *Corynebacterium glutamicum* (Merkens et al. 2005) were molecularly cloned. In the present study, *Corynebacterium efficiens* vanillate *O*-demethylase (VanAB) was overexpressed in *C. glutamicum* F (ATCC 21420), and this recombinant strain (*C. glutamicum* FVan) was used to develop a system to convert FA to PCA.

## Materials and methods

### Bacteria and media

The bacterial strains, plasmids, and oligonucleotide primers used in this study are listed in Table 1. *Escherichia coli* strains were cultured at 37 °C in Luria–Bertani (LB) medium supplemented with 50 mg/L kanamycin (Km). The phenylalanine-producing *C. glutamicum* strain ATCC 21420 was acquired from the American Type Culture Collection (ATCC). *C. efficiens* NBRC 100395 (YS-314) was acquired from the NITE Biological Resource Center (NBRC). *C. glutamicum* strains were cultured in Brain–Heart Infusion (BHI) broth (Beckton Dickinson, NJ, USA) at 30 °C, and 25 μg/mL of Km was added to the medium as appropriate.

**Table 1:** Bacterial strains, plasmids, and deoxyoligonucleotide primers

Bacterial strains, plasmids and deoxyoligonucleotide primers	Relevant characteristics or sequences	References or source
*E. coli*		
SCS110	*rpsL* (Str^r^) *thr leu endA thi*-*l lacY galK galT ara tonA tsx dam dcm*	Stratagene
	*supE44Δ* (*lac*-*proAB*) [*F’traD36 proAB lacl* ^*q*^ *ZΔM15*]	
Novablue	*endA*1 *hsdR*17 (*rK* ^−^ *mK* ^+^) *supE*44 *thi*-1 *gyrA*96 *relA*1 *lac recA*1*/F’*	Novagen
	[*proAB* ^+^ *lac I* ^*q*^ *Z ΔM*15 *Tn*10(*tet* ^*r*^)]	
*C. efficiens*		
NBRC 100395	Wild-type *C. efficiens*, YS314	NBRC
*C. glutamicum*		
ATCC 13032	Wild-type *C. glutamicum*, biotin-auxotrophic, l-glutamate producing strain	ATCC
ATCC 21420	*C. glutamicum*, l-phenylalanine producing strain	ATCC
F	*C. glutamicum* ATCC 21420 derivative harboring pCH	Okai et al. (2016)
FVan	*C. glutamicum* ATCC 21420 derivative harboring pCH-vanAB^ce^	This study
Plasmids		
pCH	*E. coli*-*C. glutamicum* shuttle vector with HCE promoter, Km^r^	Tateno et al. (2007)
pCH-vanAB^ce^	pCH containing CE0634-CE0635 (*vanAB*) from *C. efficiens* NBRC 100395	This study
Oligonucleotide primers		
CE0634_up300-F	tcattgaccgagtactccgttt	
CE0635_R	tcagggcacgtcaattgtcaggtggatatt	
BglI-CE0634_up300-F	CAACAGTTGCGCAGCCTGAATGGCtcattgaccgagtactccgttt	
BglI-CE0635_R	ATAAATCGCATTCGCCATTCAGGCTGATCGCTATTGCCCTCCGATTATTAGGAGGGCGATtcagggcacgtcaattgtcaggtggatatt	

Restriction enzyme cleavage sites are underlined, and the sequences of the primer pairs used for overlap-PCR are in small characters

### Molecular genetic procedures

The BLAST server (http://www.ncbi.nlm.nih.gov/BLAST/) was used to search genomic sequences of *C. glutamicum* and *C. efficiens* deposited in the NCBI database (http://www.ncbi.nlm.nih.gov/genome/). Genomic DNA was prepared from *C. glutamicum* strain F (ATCC 21420) grown in 5 mL of BHI medium using a Wizard Gnomic DNA Purification Kit (Promega, Madison, WI, USA). All genetic manipulations were performed using *E. coli* SCS110 to avoid DNA methylation. The *E. coli*–*C. glutamicum* shuttle vector pCH harboring the *hce* promoter to afford high-level constitutive gene expression is described in our previous study (Tateno et al. 2007). Plasmid DNAs were prepared from *E. coli* strains using the Viogene Mini Plus Plasmid DNA Extraction system (Viogene, Taipei, Taiwan).

The vanillate *O*-demethylase gene (VanAB^CE^, CE0634–CE0635) from *C. efficiens* was PCR-amplified (first reaction, 15 cycles) from *C. efficiens* NBRC 100395 (YS-314) genomic DNA using the primer pair CE0634-up300F and CE0635-R (Table 1). KOD-FX DNA polymerase (TOYOBO, Japan) was used in the PCR reactions. The 2.75-kbp fragment of CE0634-CE0635 was purified using the SV Gel and PCR Clean-Up System (Promega) and used as template for 15 cycles of a second PCR reaction using the primer pair BglI-CE0634-up300-F and BglI-CE0635-R (Table 1). The amplified 2.83-kbp fragment of CE0634-CE0635 was fused to BglI-digested pCH using the InFusion Cloning Kit (Clontech Laboratories Inc, Mountain View, CA, USA). Plasmids sequences were determined using an ABI PRISM 3130xl Genetic Analyzer (Life Technologies, CA, USA).

The plasmid pCH-vanAB^CE^ was introduced into *C. glutamicum* ATCC 21420 using a Gene Pulser XL (Bio-Rad, CA, USA) electroporator (2.5 kV, 200-μF pulses) equipped with a 0.2-cm cuvette. The cells were heat-shocked at 46 °C for 6 min. The cells were then incubated at 30 °C for 1.5 min and spread on a BHI plate containing 25 μg/mL Km. The Km-resistant *C. glutamicum* transformants were confirmed using PCR with the primers CE0634-up300F and CE0635-R (Table 1). The selected strain *C. glutamicum* ATCC 21420 (pCH-VanAB^CE^) was designated *C. glutamicum* FVan. The construction of *C. glutamicum* F strain ATCC 21420 harboring pCH is described in our previous study (Okai et al. 2016).

#### Fermentation of FA, VAN, and VA

*Corynebacterium glutamicum* F was cultured in 5 mL of BHI medium for 24 h at 30 °C. AR medium (Kurusu et al. 1990) was used for biotransformation of FA and VAN. The preculture BHI medium (0.1 mL) was added to 5 mL each of AR medium containing 2 mM FA or VAN and cultured at 30 °C for 72 h with agitation at 180 rpm. For biotransformation of FA, *C. glutamicum* strains F and FVan were independently cultured in BHI medium at 30 °C for 24 h, and 0.5 mL of cultures were transferred to 25 mL of AR medium containing 2.5 mM of VA. Cells were cultured at 30 °C for 48 h with agitation at 180 rpm. Samples (1.0 mL) of the culture supernatant were collected every 24 h and centrifuged at 10,000 rpm for 3 min at 4 °C using a KUBOTA model 3740 centrifuge (Kubota, Japan). Optical density measured at 600 nm (OD_600_) was measured simultaneously.

### Biotransformation of FA to PCA

*Corynebacterium glutamicum* FVan and *C. glutamicum* F were individually cultured in 5 mL of BHI medium for 24 h at 30 °C. AF medium, AR medium without metal ions to avoid chelating PCA, and contains FA, were used for biotransformations. Preculture solutions (0.6 mL) were added to 30 mL of AF medium containing 5 mM FA (stock solution, 200 mM FA in ethanol) in a 200-mL baffled flask. The bacteria were cultured at 30 °C for 72 h with agitation at 180 rpm using a BioShaker G-BR-200 (TAITEC, Japan). Samples (1.0 mL) of the culture supernatant were collected every 24 h.

#### Fed-batch biotransformation of FA

*Corynebacterium glutamicum* strains FVan and F were individually cultured in 5 mL of BHI medium containing Km for 24 h at 30 °C. Preculture solutions (4 mL) were added to 400 mL of BY medium [10 g peptone, 10 g meat extract, 5 g yeast extract and 5 g sodium chloride, per litter (Katsumata et al. 1984)] containing Km in a 1-L baffled flask and cultured at 30 °C for 24 h with agitation at 180 rpm. The cells (wet weight, 1.2 g) were collected by centrifugation at 12,400×*g* for 15 min at 4 °C using a KUBOTA model 7780 centrifuge (Kubota). Y medium (20 g peptone, 5 g yeast extract, 5 g sodium chloride, 0.2 mg biotin, and 0.2 mg thiamine per L) was used for the biotransformation of FA. The cells were suspended in 5 mL of Y medium and added to 55 mL of Y medium containing 25 μg/mL Km in 0.2-L vessels. The fed-batch biotransformation of FA was performed using a MiniJar 8 ^MICROBIO^ (ABLE, Corp., Tokyo, Japan) for 12 h at 30 °C. The medium was maintained at pH 7.5, and the culture vessel was rotated at 400 rpm. Aeration was maintained at 0.2 vvm. The fed-batch transformation of FA started by adding FA to a final concentration of 5 mM (stock solution, 1 M FA in DMSO) to the medium and then 4 and 8 h later.

#### Analysis of aromatic compounds

PCA, FA, VAN, and VA were purchased from Wako Chemicals (Tokyo, Japan). The culture supernatants were filtered using a TRAST Disc Syringe Filter (0.45 μm, I.D. 13 mm, nylon membrane; Shimadzu GLC Ltd.) before high-performance liquid performance chromatography (HPLC) analysis. The concentrations of PCA, FA, VAN, and VA in the supernatants were determined using a Prominence high-performance liquid performance chromatography system (Shimadzu, Kyoto, Japan). A COSMOSIL Cholester column (5 μm, 150 mm × 4.6-mm I.D.) (Nacalai Tesque, Tokyo, Japan) was used to analyze PCA. The mobile phase (20% [v/v] methanol, 0.1% formate [v/v]) was delivered at 1.0 mL/min, and the column was maintained at 30 °C. A COSMOSIL 5C18-MS-II column (5 μm, 150 mm × 4.6-mm I.D.) (Nacalai Tesque) was used to analyze FA, VAN, and VA. The mobile phase (10% [v/v] methanol in 50 mM sodium phosphate buffer, pH 7.0) was delivered at 1.0 mL/min, and the column was maintained at 30 °C. PCA, FA, VAN, and VA were detected at 254 nm using a Prominence SPD-10A (Shimadzu).

#### Nucleotide sequence analysis of genomic DNA

The genomic DNA of *C. glutamicum* ATCC 21420 was purified using a Wizard Genomic DNA Purification Kit (Promega) and sequenced using a Next Generation Sequencing (NGS) system as follows: The DNA was fragmented using a Biorupter UCD-200TS Sonicator System (Diagenote, NJ, USA) at 4 °C to yield 0.5–1.0 kb fragments. Fragment size was evaluated using an Agilent 2100 Bioanalyzer (Agilent Technologies, CA, USA), and the purities of the fragments were determined using a Bioanalyzer HS DNA Kit, a Qubit dsDNA HS Assay Kit (Invitrogen), and a Qubit 2.0 Fluorometer (Invitrogen). Construction of a genomic DNA library for sequencing was performed using an UltraDNA Library Prep Kit for Illumina (New England Biolabs, MA, USA) according to the manufacturer’s instructions. NEBNext Multiplex Oligos for Illumina (Index Primers Set1, New England Biolabs) were used to ligate adaptors to the DNA fragments. Paired-end sequencing was performed using the MiSeq Reagent Kit v3 and MiSeq Sequencing Systems (Illumina). Sequence data were analyzed using the CLC Bio Genomic Workbench 7.0. (CLC Bio, Germany). The sequences of strain ATCC 21420 were mapped to the reference genome sequence (NC_003450) of *C. glutamicum* type strain (ATCC13032). The nucleotide sequence was analyzed using the BLAST server (http://www.ncbi.nlm.nih.gov/).

#### Cell growth assay

Cell growth assays in PCA medium were performed using *C. glutamicum* strains F and W, which were grown on 5 mL of BHI medium for 24 h at 30 °C. BT minimal medium (Kurusu et al. 1990) containing 2 mM PCA was used for the cell growth assays. The starter cultures (0.1 mL each) were inoculated into 5 mL of BT-2 mM PCA medium. The two strains were cultivated at 30 °C with agitation at 60 rpm for 24 h. Optical density at 660 nm was monitored once hourly using a TVS062CA Bio-Photorecorder (ADVANTEC Toyo, Tokyo, Japan).

## Results

### Biotransformation of FA and VAN

*Corynebacterium glutamicum* ATCC 13032 utilizes FA, VAN, and VA as carbon sources (Merkens et al. 2005) and catabolizes PCA via two pathways. Thus, FA can be catabolized through VAN and VA to PCA via a non-β-oxidative, catabolic pathway (Merkens et al. 2005) (Fig. 1-I) as well through the β-oxidative, *phd* pathway via VA (Kallscheuer et al. 2016) (Fig. 1-II). PCA is then converted to succinyl-CoA and acetyl-CoA through the β-ketoadipate pathway (Brinkrolf et al. 2006).

**Fig. 2:**
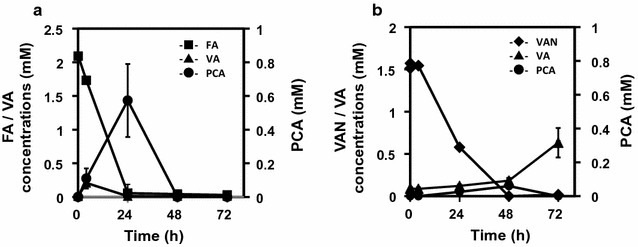
Transformation assays of FA and VAN. *C. glutamicum* strain F was cultured in BHI medium at 30 °C for 24 h, and 0.1 mL of each culture was transferred to 5 mL of AR medium containing 2 mM each of FA or VAN with 25 μg/mL of Km in 5 mL tubes. The biotransformation of **a** FA and **b** VAN was performed at 30 °C with agitation at 180 rpm for 72 h. The concentrations of extracellular FA (*squares*), VA (*triangles*), VAN (*diamonds*) and PCA (*circles*) were monitored every 24 h. Data represent the mean and standard error from three independent experiments

The aromatic amino acid-producing strain *C. glutamicum* ATCC 21420 has the ability to synthesize extracellular PCA when grown on glucose (Okai et al. 2016), and therefore its ability to metabolize FA and VAN was determined here. *C. glutamicum* F (ATCC 21420) harboring pCH was cultured in AR medium containing 2 mM FA or VAN, and extracellular PCA was analyzed (Fig. 2). *C. glutamicum* F utilized 2 mM each of FA and VAN within 48 h. Formation of 0.59 ± 0.19 mM of extracellular PCA was detected in the medium containing 2.09 ± 0.02 mM of FA after 24 h (Fig. 2a), and only 0.06 mM PCA was detected in the medium after 48 h when 1.51 ± 0.03 mM of VAN was added to the medium (Fig. 2b). Extracellular VA was detected 4 h after incubation with FA (Fig. 2a).

### Biotransformation of VA to PCA by *C. glutamicum* F expressing *C. efficiens vanAB*

**Fig. 3:**
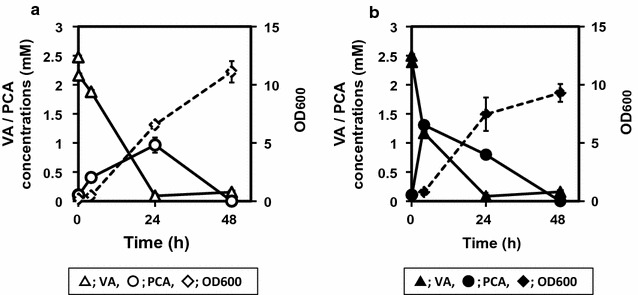
Biotransformation of VA to PCA by *C. glutamicum* expressing vanillate *O*-demethylase (Van). **a** *C. glutamicum* strain F (*open symbols*) as a control and **b** strain FVan (*closed symbols*) were cultured in BHI medium at 30 °C for 24 h, and 0.5 mL of each culture was transferred to 25 mL of AR medium containing 2.5 mM VA. The bioconversion was performed at 30 °C for 48 h with agitation at 180 rpm. Extracellular VA (*triangles*) and PCA (*circles*) were analyzed every 24 h. OD_600_ (*diamonds*) was monitored simultaneously. Data represent the mean and standard error of three independent experiments

The conversion of VA to PCA is the last step in the transformation of FA to PCA via the two catabolic pathways described above (Fig. 1-I, II). Extracellular VA and PCA were detected in cultures of *C. glutamicum* F containing FA. We overexpressed vanillate *O*-demethylase (VanAB) to increase the production of PCA from FA. The *C. efficiens* NBRC 100395 (YS-314) vanillate *O*-demethylase gene (*van*AB^CE^; CE0634–CE0635) was expressed in *C. glutamicum* ATCC 21420 to generate strain FVan. To investigate the expression of VanAB by *C. glutamicum* FVan, biotransformation of VA was performed using *C. glutamicum* strains FVan and F, which were individually cultured in AR medium containing 2.5 mM VA (Fig. 3). Strain F consumed 2.47 ± 0.01 mM of VA and produced 0.96 mM of PCA after 24 h (Fig. 3a). Strain FVan consumed 2.48 ± 0.01 mM of VA and produced a maximum of 1.30 ± 0.03 mM extracellular PCA after 4 h (Fig. 3b). When 2.5 mM of VA was added to the medium, the growth of strain FVan was similar to that of strain F (Fig. 3a, b).

### Biotransformation of FA to PCA by *C. glutamicum* strains FVan and F

**Fig. 4:**
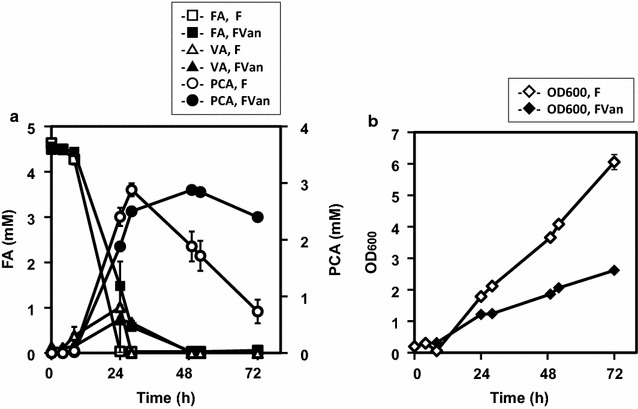
Biotransformation of FA to PCA by recombinant strains of *C. glutamicum*. *C. glutamicum* strains FVan (*closed symbols*) and F (*open symbols*) as a control were cultured in BHI medium at 30 °C for 24 h, and 0.5 mL of each culture was transferred to 25 mL of AF medium containing 5 mM FA and 25 μg/mL Km in 200-mL baffled flasks. The biotransformation of FA was performed at 30 °C with agitation at 180 rpm for 72 h. **a** Concentrations of extracellular FA (*squares*), VA (*triangles*), and PCA (*circles*) were monitored every 24 h. **b** OD_600_ (*diamonds*) was monitored simultaneously. Data represent the mean and standard error of three independent experiments

*Corynebacterium glutamicum* strains FVan and F were cultivated in BHI medium for 24 h, and 0.5 mL of the culture was transferred to 25 mL of AF medium containing 5 mM FA. Biotransformation was performed at 30 °C with agitation at 180 rpm for 72 h (Fig. 4). FA in the medium markedly decreased after 24 h, and extracellular PCA was detected in cultures of strains F and FVan. Strain FVan produced 2.87 ± 0.01 mM (442.2 mg/L) of extracellular PCA (maximum at 48 h) from 4.57 ± 0.01 mM FA, and the molar conversion yield was 62.8% (mol/mol) (Fig. 4a). Strain F produced extracellular PCA from 4.63 ± 0.01 mM of FA, with a maximum of 2.88 ± 0.11 mM after 28 h. Extracellular PCA decreased in cultures of strain F after 28 h of cultivation. Thus, strain FVan produced extracellular PCA from FA more stably compared with strain F in 48 h (Fig. 4a). The growth of strain FVan was slower compared with that of strain F in the medium containing FA (Fig. 4b). During the biotransformation of FA by both strains, the production of VA peaked at 24 h. The concentrations of extracellular VA in cultures of strains FVan and F were 1.47 and 0.81 mM after 24 h, respectively (Fig. 4a).

### Fed-batch transformation of FA by *C. glutamicum* strains FVan and F

**Fig. 5:**
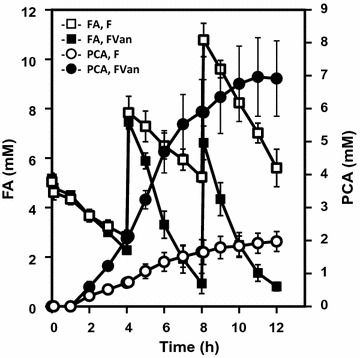
Fed-batch biotransformation of FA. *C. glutamicum* strains FVan (*closed symbols*) and F (*open symbols*) were cultivated in BY medium in a flask for 24 h, collected, and inoculated into a bioreactor. The cells (wet weight 1.2 g) were collected and transferred to 60 mL of Y medium. The fed-batch biotransformation of FA started with agitation at 400 rpm at 30 °C and the addition of 5 mM FA. The medium was maintained at pH 7.0. FA (5 mM) was added to the medium after 4 and 8 h. OD_600_ was monitored simultaneously. Data represent the mean and standard error of three independent experiments

To increase the rate of transformation of FA to PCA, fed-batch biotransformation of FA was performed using strains FVan and F using a bioreactor for 12 h (Fig. 5). Fed-batch biotransformation of FA, performed using 1.2 g wet weight of cells, was started by adding 5 mM of FA to the Y medium, and 5 mM of FA was added after 4 and 8 h. The reaction was performed at 30 °C at pH 7.5 with agitation at 400 rpm. Strain FVan produced 6.91 ± 1.14 mM (1064.8 mg/L) of extracellular PCA from 16.0 mM of FA added to the medium within 12 h (Fig. 5). The conversion yield of FA to PCA by strain FVan was 43.5% (mol/mol) after 12 h. The maximum rate of conversion of FA to PCA by strain FVan was 0.78 mM/h (120.4 mg/L/h) after 6 h, and strain F produced 1.97 ± 0.30 mM of PCA from 15.5 mM of FA after 12 h (Fig. 5).

### Comparative sequence analysis of *pca* clusters

To understand why *C. glutamicum* ATCC 21420 efficiently produced extracellular PCA, NGS analysis was performed. We sequenced 1,160,511,997 bases with an average length = 446.9 bp of paired-end reads. The genomic sequence of strain ATCC 21420 was mapped to that of *C. glutamicum* type strain ATCC 13032 (NC_003450, genome = 3.30 Mbp). The 1,002,849,328 bases of mapped reads achieved 303.0-fold coverage. The genes encoding the enzymes required for the cleavage and utilization of aromatic compounds such as PCA and PHBA (β-ketoadipate pathway: *pca* gene cluster, *pcaKpobA, pcaHGBC, pcaRFDO,* and *pcaIJ*) (Additional file 1: Figure S1) were conserved in the genome of *C. glutamicum* ATCC 21420 (Additional file 2: Table S1).

Comparative sequence analysis of *pca* genes of strain ATCC 21420 vs strain ATCC 13032 revealed that the *pca* genes were oriented in the same order. Missense mutations were detected in the *pca* genes of strain ATCC 21420 (Additional file 2: Table S1). Notably, in strain F, insertions were identified in the *pcaK* gene (Additional file 2: Table S1), which catalyzes the import of extracellular PCA (Chaudhry et al. 2007). In strain F, the missense mutations that changed amino acid residues were detected in *pcaD* (β-ketoadipate enol-lactone hydrolase)*, pcaC* (γ-carboxymuconolactone decarboxylase), *pcaB* (β-carboxy-cis,cis-muconate cycloisomerase), and *pcaGH* (protocatechuate 3,4-dioxygenase), which cleave aromatic rings and catalyze the formation of γ-carboxy-muconolactone and β-ketoadipate enol-lactone. In contrast, the predicted amino acid sequences of *pcaIJ* (β-ketoadipate succinyl-CoA transferase) and *pcaF* (β-ketoadipyl-CoA thiolase), which catalyze reactions of β-ketoadipate with acetyl-CoA and succinyl-CoA, were identical (Additional file 2: Table S1). The *vanAB* gene was present in *C. glutamicum* strain ATCC 21420, and its predicted amino acid sequence was identical to that of VanAB (Cgl2383-2384) of *C. glutamicum* ATCC 13032.

### Cell growth assays

Cell growth assays in PCA medium were performed using *C. glutamicum* strains F and W (Additional file 3: Figure S2), which were individually cultured in minimal BT medium containing 2 mM PCA for 24 h. Although strain W grew in BT-2 mM PCA, strain F did not for the first 10 h and grew slowly after 15 h (Additional file 3: Figure S2).

## Discussion

Extracellular PCA was produced from FA by *C. glutamicum* F expressing *C. efficiens* vanillate *O*-demethylase. *C. glutamicum* strain FVan transformed 4.57 mM of FA into 2.87 mM of extracellular PCA, maximum conversion rate = 62.8% (mol/mol) after 48 h (Fig. 4). In the fed-batch biotransformation of FA, *C. glutamicum* FVan produced 6.9 mM (1064 mg/L) of PCA from 16.0 mM of FA. The conversion rate of FA to PCA by strain FVan reached 0.78 mM/h (120 mg/L/h) after 6 h of fed-batch biotransformation (Fig. 5). To the best of our knowledge, this is the highest rate achieved for the conversion of FA to PCA by *C. glutamicum* or other microorganisms. Further, strain F converted FA to PCA (Figs. 4, 5).

Some strains of *Pseudomonas* species convert FA to PCA (Venturi et al. 1998; Priefert et al. 1997). For example, *P. putida* WCS 358 catabolizes FA to VAN, VA, and PCA (Venturi et al. 1998). *E. coli* harboring the vanillin dehydrogenase (*Vdh*) gene from *Pseudomonas* HR199 converts 14 mM of VAN to 13 mM of VA and 0.5 mM of PCA (Priefert et al. 1997). Wild-type *C. glutamicum* catabolizes FA, VAN, and VA as carbon sources (Merkens et al. 2005; Brinkrolf et al. 2006). However, to the best of our knowledge, biotransformation of FA to produce PCA by these microorganisms has not been reported.

In *C. glutamicum* F, PCA was produced mainly from FA (Fig. 2a), and only a small amount of PCA was formed from VAN (Fig. 2b). Therefore this strain likely converts FA to VA via the *phd* pathway (Fig. 1-II). The genome sequence of strain ATCC 21420 harbors predicted inactivating mutations of the PCA importer (*pcaK*) (Additional file 2: Table S1), consistent with the amount of extracellular PCA formed by this strain (Figs. 4, 5). Strain F converted FA to PCA in the medium, therefore it will be applicable for the conversion of various lignin-derived aromatic compounds to PCA. Although slow growth was observed in BT-2 mM PCA medium, strain F mainly lacked the ability to utilize PCA compared with strain W (Additional file 3: Figure S2).

PCA was slowly degraded by strain F during the catabolism of FA (Fig. 4a). By preventing PCA degradation by strain F, PCA production will be improved. In strain F, certain mutations were detected in *pca*D (β-ketoadipate enol-lactone hydrolase), *pca*C (γ-carboxymuconolactone decarboxylase), *pca*B (β-carboxy-cis,cis-muconate cycloisomerase), and *pca*GH (protocatechuate 3,4-dioxygenase), which mediate the cleavage of the aromatic rings of PCA through the formation of γ-carboxy-muconolactone (Additional file 2: Table S1). We are also attempting genomic sequence analysis of strain F to search for another PCA degradation route. Strain FVan produced PCA stably as VA was converted to PCA (Fig. 4a).

*Corynebacterium glutamicum* ATCC 21420 (strain F) produces phenylalanine (Okumura et al. 1972). Extracellular PCA (1168.1 ± 27 mg/L) was produced from glucose by *C. glutamicum* F expressing the *E. coli* CPL gene *ubiC*, and strain F produces extracellular PCA (Okai et al. 2016). *C. glutamicum* FVan converts FA to PCA, and it will therefore be suitable for the cost-effective production of PCA from biomass-FA for use as a precursor to biomass-based plastics. The production of biomass-plastics will be realized when methods are developed to extract PCA from the fermentation broth.

PCA also has the potential for use in functional foods and pharmaceuticals. PCA contributes to the control of oxidative stress and inflammation in mammals (Masella et al. 2012). For example, the antioxidant and anti-inflammatory effects of PCA treatment were analyzed in vitro and in vivo (Semaming et al. 2015). PCA possess antibacterial and antiviral activities (Kakkar and Bais 2014). For example, PCA inhibits avian influenza virus infection of birds (Ou et al. 2014). PCA treatment reduces the inflammation of lung cells in mice and suppresses the inflamed cells (Ou et al. 2014). *C. glutamicum* is a generally recognized as safe microorganism and is used in industry to produce amino acids for food. Therefore, it will be suitable for fermentation of PCA for producing food additives, precursors of pharmaceuticals, and bioplastics.

In the current study, we show that *C. glutamicum* ATCC 21420 utilized lignin-derived phenolic compounds (FA, VA) to produce PCA. Notably, a platform for the biotransformation of FA to PCA using *C. glutamicum* was established here. Our future course focuses on producing PCA from lignocellulosic plant biomass, in which FA is esterified to the arabinosyl residue in arabinoxylan (Gopalan et al. 2015). FA can be extracted using ferulic acid esterases (FAEs) from plant biomass (Gopalan et al. 2015). FA is extracted from wheat bran using *Penicillium funiculosum* FAE (Kroon et al. 2000), from corn bran using *Neosartoryaspinosa* crude FAE (Shin et al. 2006), and from wheat arabinoxylan using *Aspergillus clavatus* FAE (Damasio et al. 2013). Further, FA is extracted from sugar beet pulp using a crude extract of *Aspergillus niger* (Bonnin et al. 2002) and from autoclaved maize bran using *A. niger* FAE (Benoit et al. 2006). Heterologous expression of these FAEs in *C. glutamicum* will serve as a promising approach for extracting FA from plant biomass. Moreover, we plan to express xylanases in *C. glutamicum*. For the utilization of cellulosic materials, our expression system of *C. glutamicum* that expresses endoglucanase (Tsuchidate et al. 2011) will be applicable as well.

In conclusion, *C. glutamicum* ATCC 21420 (F) utilizes lignin-derived phenolic compounds (FA and VA) and produces PCA. Biotransformation of FA to PCA using *C. glutamicum* was established in the present study. *C. glutamicum* ATCC 21420, which expressed *C. efficiens* vanillate *O*-demethylase, converted FA to PCA with a maximum conversion yield of 62.8% (mol/mol).

## Additional files

**Additional file 1: Figure S1.** Catabolic pathway and PCA import system of *Corynebacterium glutamicum*. The reactions of the beta-ketoadipate pathway and PCA transporter in *C. glutamicum*. 4-Hydroxybenzoic acid (HBA) is converted to PCA by the reaction of 4-HBA hydroxylase (PobA). Extracellular PCA is imported by PcaK in the type strain. PCA is then catabolized to the TCA cycle intermediates acetyl-CoA and succinyl-CoA via the β-ketoadipate pathway catalyzed by PCA enzymes.

**Additional file 2: Table S1.** Mutations of *pca* genes in *C. glutamicum* ATCC 21420 compared with *C. glutamicum* ATCC 13032.

**Additional file 3: Figure S2.** Growth of *C. glutamicum* strains F and W in BT-PCA medium. *C. glutamicum* strains F (closed symbols) and W (open symbols) were grown in 5 mL of BHI medium for 24 h at 30℃. Each starter culture (0.1 mL) was inoculated into 5 mL of BT-2mM PCA medium. The two strains were cultured at 30℃ with agitation at 60 rpm for 24 h. The optical densities at 660nm were monitored once hourly. The data represent the mean and standard error of three independent experiments.

## Abbreviations

PCA: protocatechuic acid (3,4-dihydroxybenzoic acid)
FA: ferullic acid (*trans*-4-hydroxy-3-methoxycinnamic acid)
VAN: vanillin (4-hydroxy-3-methoxybenzoaldehyde)
VA: vanillic acid (4-hydroxy-3-methoxybenzoic acid)
CoA: coenzymeA
Van: Vanillate 3-*O*-demethylase
