# Supplementary material for: Biotransformation of ferulic acid to protocatechuic acid by Corynebacterium glutamicum ATCC 21420 engineered to express vanillate O-demethylase
Source: AMB Express. 2017 Jun 21;7:130. doi: 10.1186/s13568-017-0427-9 (PMC5479773; doi:10.1186/s13568-017-0427-9)
Supplement: Supplementary file 1 — Additional file 1: Figure S1. Catabolic pathway and PCA import system of Corynebacterium glutamicum. The reactions of the beta-ketoadipate pathway and PCA transporter in C. glutamicum. 4-Hydroxybenzoic acid (HBA) is converted to PCA by the reaction of 4-HBA hydroxylase (PobA). Extracellular PCA is imported by PcaK in the type strain. PCA is then catabolized to the TCA cycle intermediates acetyl-CoA and succinyl-CoA via the β-ketoadipate pathway catalyzed by PCA enzymes. [file 13568_2017_427_MOESM1_ESM.docx]

Supplementary material

**AMB Express**

**Biotransformation of ferulic acid to protocatehuic acid by *Corynebacterium glutamicum* ATCC21420 engineered to express vanillate *O*-demethylase*.***

Naoko Okai^1^, Takaya Masuda^2^, Yasunobu Takeshima^1^, Kosei Tanaka^3^, Ken-ichi Yoshida^1^, Masanori Miyamoto^2^, Chiaki Ogino^4^, and Akihiko Kondo^1,5、＊^

^1^ Graduate School of Science, Technology, and Innovation, Kobe University, 1-1 Rokkodaicho, Kobe 657-8501, Japan

^2^ Raw Materials and Polymers Technology Department, Raw Materials and Polymers Division, Teijin Limited, 2345 Nishihabu-cho, Matsuyama-shi, Ehime, 791-8536, Japan

^3^ Organization of Advanced Science and Technology, Kobe University, 1-1 Rokkodaicho, Kobe 657-8501, Japan

^4^ Department of Chemical Science and Engineering, Graduate School of Engineering, Kobe University, 1-1 Rokkodaicho, Kobe 657-8501, Japan

^5^ Biomass Engineering Program, RIKEN, 1-7-22 Suehiro-cho, Tsurumi-ku, Yokohama 230-0045, Japan

* Corresponding author; Akihiko Kondo

E-mail : akondo@kobe-u.ac.jp (A. Kondo)

Tel: +81-78-803-6196; Fax: +81-78-803-6196


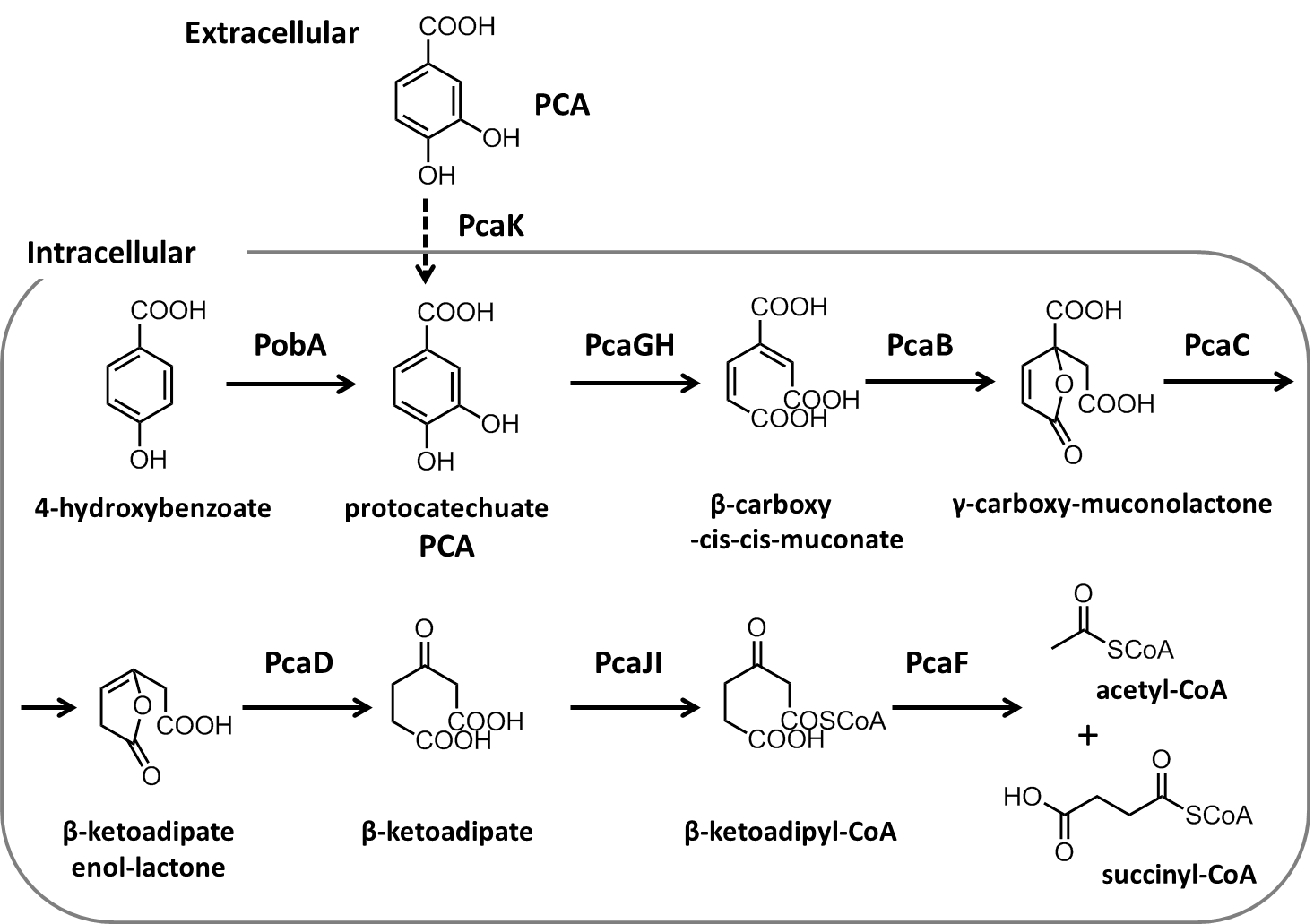


**Figure S1. Catabolic pathway and PCA import system of *Corynebacterium glutamicum***.

The reactions of the beta-ketoadipate pathway and PCA transporter in *C. glutamicum*. 4-Hydroxybenzoic acid (HBA) is converted to PCA by the reaction of 4-HBA hydroxylase (PobA). Extracellular PCA is imported by PcaK in the type strain. PCA is then catabolized to the TCA cycle intermediates acetyl-CoA and succinyl-CoA via the β-ketoadipate pathway catalyzed by PCA enzymes.
