# Supplementary material for: Biotransformation of ferulic acid to protocatechuic acid by Corynebacterium glutamicum ATCC 21420 engineered to express vanillate O-demethylase
Source: AMB Express. 2017 Jun 21;7:130. doi: 10.1186/s13568-017-0427-9 (PMC5479773; doi:10.1186/s13568-017-0427-9)
Supplement: Supplementary file 2 — Additional file 2: Table S1. Mutations of pca genes in C. glutamicum ATCC 21420 compared with C. glutamicum ATCC 13032. [file 13568_2017_427_MOESM2_ESM.docx]

Supplementary material

**AMB Express**

**Biotransformation of ferulic acid to protocatehuic acid by *Corynebacterium glutamicum* ATCC21420 engineered to express vanillate *O*-demethylase*.***

Naoko Okai^1^, Takaya Masuda^2^, Yasunobu Takeshima^1^, Kosei Tanaka^3^, Ken-ichi Yoshida^1^, Masanori Miyamoto^2^, Chiaki Ogino^4^, and Akihiko Kondo^1,5＊^

^1^ Graduate School of Science, Technology, and Innovation, Kobe University, 1-1 Rokkodaicho, Kobe 657-8501, Japan

^2^ Raw Materials and Polymers Technology Department, Raw Materials and Polymers Division, Teijin Limited, 2345 Nishihabu-cho, Matsuyama-shi, Ehime, 791-8536, Japan

^3^ Organization of Advanced Science and Technology, Kobe University, 1-1 Rokkodaicho, Kobe 657-8501, Japan

^4^ Department of Chemical Science and Engineering, Graduate School of Engineering, Kobe University, 1-1 Rokkodaicho, Kobe 657-8501, Japan

^5^ Biomass Engineering Program, RIKEN, 1-7-22 Suehiro-cho, Tsurumi-ku, Yokohama 230-0045, Japan

* Corresponding author; Akihiko Kondo

E-mail : akondo@kobe-u.ac.jp (A. Kondo)

Tel: +81-78-803-6196; Fax: +81-78-803-6196

| **Table S1 Mutations of *pca* genes in *C. glutamicum* ATCC 21420 compared with *C. glutamicum* ATCC 13032.** | | | | | | |
| --- | --- | --- | --- | --- | --- | --- |
| **Reference nucleotide position** | **Gene** | **Annotation** | **Reference gene** | **Coding region (amino acid)** | **Mutation** | **Amino acid change** |
| 1123531 | *pobA* | 4-hydroxybenzoate 3-monooxygenase | Cgl1032 | 431 | Missense | Ser307Ala |
|  |  |  |  |  |  | Pro236Ser |
| 1124833 | *pcaK* | protocatechuic acid transporter, | Cgl1031 | 395 | Insertion | 982InsAC |
|  |  | major facilitator superfamily permease |  |  | Missense | Ser235Phe |
|  |  |  |  |  | Missense | Leu182Ile |
|  |  |  |  |  | Insertion | 183InsLL |
| 2531967 | *pcaJ* | acyl-CoA:acetate CoA transferase beta subunit | Cgl2389 | 211 | Missense | none |
| 2532602 | *pcaI* | acyl-CoA:acetate CoA transferase alpha subunit | Cgl2390 | 250 | Missense | none |
| 2533419 | *pcaR* | *pca* regulon regulatory protein | Cgl2391 | 255 | Missense | Ile154Val |
|  |  |  |  |  |  | Ala185Thr |
| 2534202 | *pcaF* | acetyl-CoA acetyltransferase | Cgl2392 | 408 | Deletion and Insertion | 216_217delTCinsCT |
|  |  |  |  |  | Missense | Ile113Val |
| 2535451 | *pcaD* | 3-oxoadipate enol-lactonase | Cgl2393 | 251 | Missense | Asn80Ser |
|  |  |  |  |  |  | Glu117Ala |
|  |  |  |  |  |  | Pro168Ser |
| 2536197 | *pcaO* | DNA-binding HTH domain-containing protein | Cgl2394 | 687 | Missense | His328Leu |
|  |  |  |  |  |  | Ser412Phe |
| 2538246 | *pcaC* | 4-carboxymuconolactone decarboxylase | Cgl2395 | 122 | Missense | Ala18Thr |
| 2538614 | *pcaB* | 3-carboxy-cis,cis-muconate cycloisomerase | Cgl2396 | 372 | Missense | Ser16Pro |
|  |  |  |  |  |  | Arg198His |
|  |  |  |  |  |  | Ala346Val |
| 2539707 | *pcaG* | protocatechuate 3,4-dioxygenase, alpha subunit | Cgl2397 | 204 | Missense | Ala115Thr |
|  |  |  |  |  |  | Ile18Val |
| 2540333 | *pcaH* | protocatechuate 3,4-dioxygenase, beta subunit | Cgl2398 | 230 | Missense | Arg152His |
