# Supplementary material for: Biotransformation of ferulic acid to protocatechuic acid by Corynebacterium glutamicum ATCC 21420 engineered to express vanillate O-demethylase
Source: AMB Express. 2017 Jun 21;7:130. doi: 10.1186/s13568-017-0427-9 (PMC5479773; doi:10.1186/s13568-017-0427-9)
Supplement: Supplementary file 3 — Additional file 3: Figure S2. Growth of C. glutamicum strains F and W in BT-PCA medium. C. glutamicum strains F (closed symbols) and W (open symbols) were grown in 5 mL of BHI medium for 24 h at 30℃. Each starter culture (0.1 mL) was inoculated into 5 mL of BT-2mM PCA medium. The two strains were cultured at 30℃ with agitation at 60 rpm for 24 h. The optical densities at 660nm were monitored once hourly. The data represent the mean and standard error of three independent experiments. [file 13568_2017_427_MOESM3_ESM.pptx]

## Slide 1
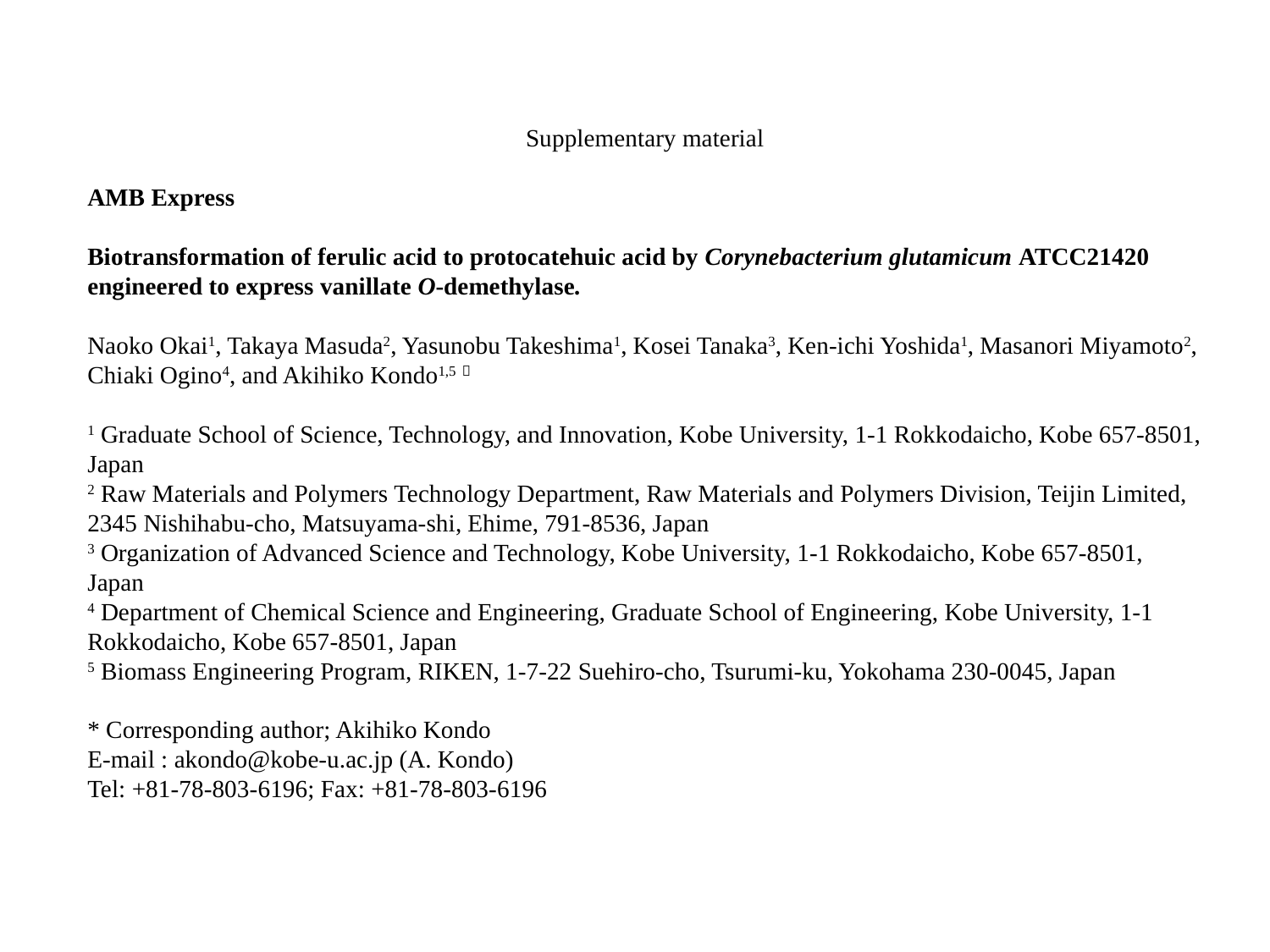

Supplementary material
AMB Express
Biotransformation of ferulic acid to protocatehuic acid by Corynebacterium glutamicum ATCC21420 engineered to express vanillate O-demethylase.
Naoko Okai1, Takaya Masuda2, Yasunobu Takeshima1, Kosei Tanaka3, Ken-ichi Yoshida1, Masanori Miyamoto2, Chiaki Ogino4, and Akihiko Kondo1,5＊
1 Graduate School of Science, Technology, and Innovation, Kobe University, 1-1 Rokkodaicho, Kobe 657-8501, Japan
2 Raw Materials and Polymers Technology Department, Raw Materials and Polymers Division, Teijin Limited, 2345 Nishihabu-cho, Matsuyama-shi, Ehime, 791-8536, Japan
3 Organization of Advanced Science and Technology, Kobe University, 1-1 Rokkodaicho, Kobe 657-8501, Japan
4 Department of Chemical Science and Engineering, Graduate School of Engineering, Kobe University, 1-1 Rokkodaicho, Kobe 657-8501, Japan
5 Biomass Engineering Program, RIKEN, 1-7-22 Suehiro-cho, Tsurumi-ku, Yokohama 230-0045, Japan
* Corresponding author; Akihiko Kondo
E-mail : akondo@kobe-u.ac.jp (A. Kondo)
Tel: +81-78-803-6196; Fax: +81-78-803-6196

## Slide 2
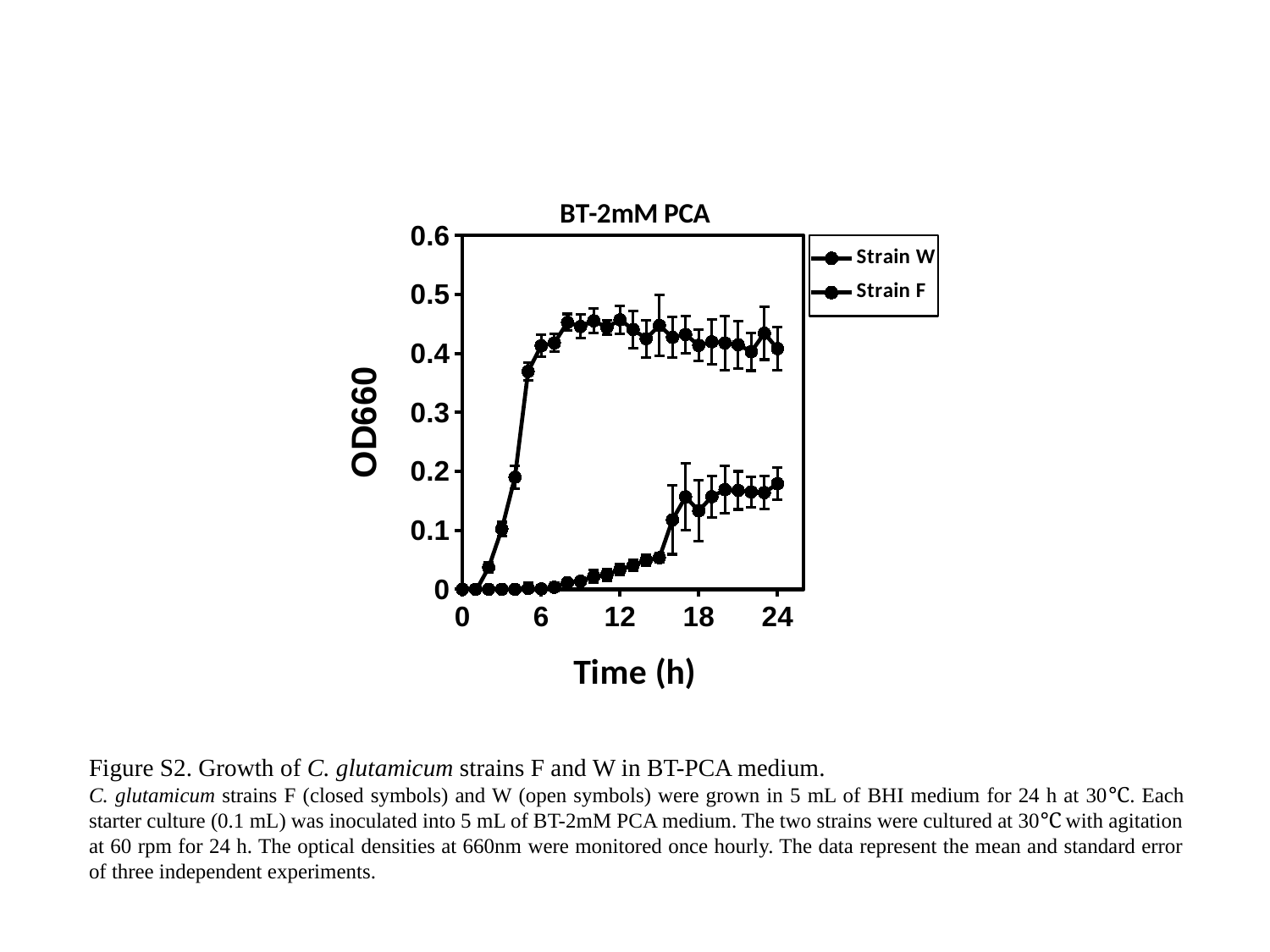

### Chart: BT-2mM PCA
| Category | | |
|---|---|---|Figure S2. Growth of C. glutamicum strains F and W in BT-PCA medium.
C. glutamicum strains F (closed symbols) and W (open symbols) were grown in 5 mL of BHI medium for 24 h at 30℃. Each starter culture (0.1 mL) was inoculated into 5 mL of BT-2mM PCA medium. The two strains were cultured at 30℃ with agitation at 60 rpm for 24 h. The optical densities at 660nm were monitored once hourly. The data represent the mean and standard error of three independent experiments.
